# Manganese-Labeled Alginate Hydrogels for Image-Guided Cell Transplantation

**DOI:** 10.3390/ijms23052465

**Published:** 2022-02-23

**Authors:** Antonina M. Araszkiewicz, Eduarda P. Oliveira, Terje Svendsen, Katarzyna Drela, Piotr Rogujski, Izabela Malysz-Cymborska, Michal Fiedorowicz, Rui L. Reis, Joaquim Miguel Oliveira, Piotr Walczak, Miroslaw Janowski, Barbara Lukomska, Luiza Stanaszek

**Affiliations:** 1 NeuroRepair Department, Mossakowski Medical Research Institute, Polish Academy of Sciences, 02-106 Warsaw, Poland; antonina.araszkiewicz@gmail.com (A.M.A.); progujski@imdik.pan.pl (P.R.); barbara.lukomska@imdik.pan.pl (B.L.); 2 3B’s Research Group, I3Bs—Research Institute on Biomaterials, Biodegradables and Biomimetics, University of Minho, Headquarters of the European Institute of Excellence on Tissue Engineering and Regenerative Medicine, 4805-017 Guimarães, Portugal; eduarda.oliveira@i3bs.uminho.pt (E.P.O.); rgreis@i3bs.uminho.pt (R.L.R.); miguel.oliveira@i3bs.uminho.pt (J.M.O.); 3 ICVS/3B’s—PT Government Associated Laboratory, 4710-057 Guimarães, Portugal; 4 FMC Biopolymer AS, 1337 Sandvika, Norway; terje.svendsen@dupont.com; 5 Medical Research Agency, 00-014 Warsaw, Poland; katarzyna.drela@abm.gov.pl; 6 Department of Neurosurgery, School of Medicine, Collegium Medicum, University of Warmia and Mazury, 10-082 Olsztyn, Poland; i.malysz-cymborska@uwm.edu.pl; 7 Small Animal Magnetic Resonance Imaging Laboratory, Mossakowski Medical Research Institute, Polish Academy of Sciences, 02-106 Warsaw, Poland; mfiedorowicz@imdik.pan.pl; 8 Program for Image Guided Neurointerventions, Department of Diagnostic Radiology and Nuclear Medicine, Center for Advanced Imaging Research, University of Maryland Marlene and Stewart Greenebaum Comprehensive Cancer Center, University of Maryland, Baltimore, MD 21201, USA; pwalczak@som.umaryland.edu (P.W.); miroslaw.janowski@som.umaryland.edu (M.J.)

**Keywords:** alginate, bio-scaffolds, central nervous system, hydrogels, manganese, MRI, stem cells

## Abstract

Cell transplantation has been studied extensively as a therapeutic strategy for neurological disorders. However, to date, its effectiveness remains unsatisfactory due to low precision and efficacy of cell delivery; poor survival of transplanted cells; and inadequate monitoring of their fate in vivo. Fortunately, different bio-scaffolds have been proposed as cell carriers to improve the accuracy of cell delivery, survival, differentiation, and controlled release of embedded stem cells. The goal of our study was to establish hydrogel scaffolds suitable for stem cell delivery that also allow non-invasive magnetic resonance imaging (MRI). We focused on alginate-based hydrogels due to their natural origin, biocompatibility, resemblance to the extracellular matrix, and easy manipulation of gelation processes. We optimized the properties of alginate-based hydrogels, turning them into suitable carriers for transplanted cells. Human adipose-derived stem cells embedded in these hydrogels survived for at least 14 days in vitro. Alginate-based hydrogels were also modified successfully to allow their injectability via a needle. Finally, supplementing alginate hydrogels with Mn ions or Mn nanoparticles allowed for their visualization in vivo using manganese-enhanced MRI. We demonstrated that modified alginate-based hydrogels can support therapeutic cells as MRI-detectable matrices.

## 1. Introduction

Tissue degeneration constitutes a severe burden for societies with increasing longevity. Thus, considerable effort is directed towards regenerative medicine, but optimal types of stem cells and microenvironments able to support their function are still being studied. Amyotrophic lateral sclerosis (ALS) is a prime example of a deadly neurodegenerative disease without a cure. It is characterized by progressive loss of motor neurons. An imbalance of trophic support has been recently suggested as an attractive therapeutic target [1,2]. Stem cell therapy might replace malfunctioning cells or support the endogenous cells in their natural niche. Mesenchymal stem cells (MSCs) are currently one of the most commonly used cells in regenerative medicine [3]. These cells are known for their paracrine, trophic, and immunomodulatory activities. Independent of their origin, MSCs secrete many growth factors and anti-inflammatory cytokines, positively influencing the endogenous environment by restoring homeostasis. Thus, MSCs may protect motor neurons (MNs) and surrounding cells, contributing to tissue regeneration processes [4,5]. The cell delivery route in neurodegenerative disorders warrants careful consideration to achieve the proper therapeutic effect of transplanted cells. The administration route should be selected to allow the cells to reach the target and support the appropriate niche. Several cell delivery options are under consideration [3,6]. One is the intra-spinal route, with the significant advantage of directly placing the cells in the desired target site, although the invasive nature of this route is a disadvantage [7]. Intravenous and intraarterial routes are convenient and minimally invasive [8,9,10]. With intravenous administration, an insufficient number of injected cells reach brain parenchyma, while the intraarterial route allows for a sufficient number of delivered cells. The latter method is adequate for injuries related to massive tissue damage caused by stroke or ischemia, where infused cells possess suitable conditions to migrate into the parenchyma. The intrathecal route is minimally invasive and relatively easy to perform; cells are deposited close to the target. However, cell migration from cerebrospinal fluid (CSF) to the spine parenchyma has not been well described [7].

Another crucial aspect involves the survival of exogenous cells in the host milieu after their transplantation. Transplanted cells are subjected to mechanical forces during the injection procedure and placed in a different, frequently hostile recipient environment. Moreover, injected cells need to survive in a new niche and migrate to reach the desired target structures, which may be challenging in some areas such as intrathecal space or cerebral ventricles, where the CSF circulation might hinder the settlement process. To overcome these pitfalls, presently much effort is being directed into engineering hydrogels to serve as bio-scaffolds that can protect the cells from damaging forces during the injection procedure. This would create a protective artificial extracellular matrix for transplanted cells [11]. The hydrogels, however, must exhibit several characteristics. For example, they should possess an adequate permeability to enable the cells to exchange nutrients and metabolites, and display biocompatibility and biodegradability. Moreover, since one of the difficulties during transplantation is the post-surgical localization of the graft, it also is desirable for the hydrogel to be detectable by magnetic resonance imaging (MRI), allowing visualization in vivo [12].

Appropriate contrast must be applied to the graft to allow for visualization while not negatively affecting cell functions. Our recent studies proved manganese ions to be a proper MRI contrast agent for directly visualizing Gellan gum-based hydrogels after intrathecal transplantation [13]. Manganese exhibits similar paramagnetic properties as gadolinium; however, it is much less toxic in low concentrations. Another advantage of using manganese ions is their lack of accumulation and the possibility of tuning their clearance from the organism following release from the scaffold [14]. Thus, the candidate hydrogels should comprise a universal bio-scaffold that would serve as a simple matrix and provide protection for the cells from mechanical forces and the hostile environment of the recipient. An additional advantage is the ability to visualize transplanted material and improve its properties by supplementing it with various substances.

Alginates were previously considered scaffolds or drug delivery systems for nerve tissue engineering [15,16,17,18]. Moreover, alginate hydrogels were shown to enhance the survival of transplanted cells and improve outcomes in animal models of cardiac infarction [19], hind limb ischemia [20], and spinal cord injury [21]. Thus, alginate-based hydrogels are appealing for cell delivery due to their natural origin, biocompatibility, their resemblance of extracellular matrix, and their relatively easy manipulation with thickening processes using calcium ions [22,23].

In this study, we developed an alginate hydrogel-based bio-scaffold for stem cell delivery and manganese-enhanced magnetic resonance imaging (MEMRI). We proposed blending diverse low viscosity sodium alginates (LVM) and calcium alginate (CaM) solutions with the addition of manganese ions to create multifunctional bio-scaffolds for intrathecal stem cell delivery. Our work demonstrates the possible use of alginate hydrogel scaffolds as cell delivery carriers for the therapy of neurological and other disorders.

## 2. Results

To increase text readability, we have used simplified hydrogel naming as follows:

- Standard (SD): 1.5% LVM (Low Viscosity Sodium Alginate) + 0.5% CaM (Calcium Alginate);
- Enhanced (EN): 1.5% LVM + 1% CaM;
- Standard + 10% CB (compact beads): 1.5% LVM + 0.5% CaM + 10% CB;
- Standard + 0.1 mM Mn: 1.5% LVM + 0.5% CaM + 0.1 mM MnCl_2;_
- Enhanced + 10% CB: 1.5% LVM + 1% CaM + 10% CB.

### 2.1. Rheology of LVM

**Figure 1:**
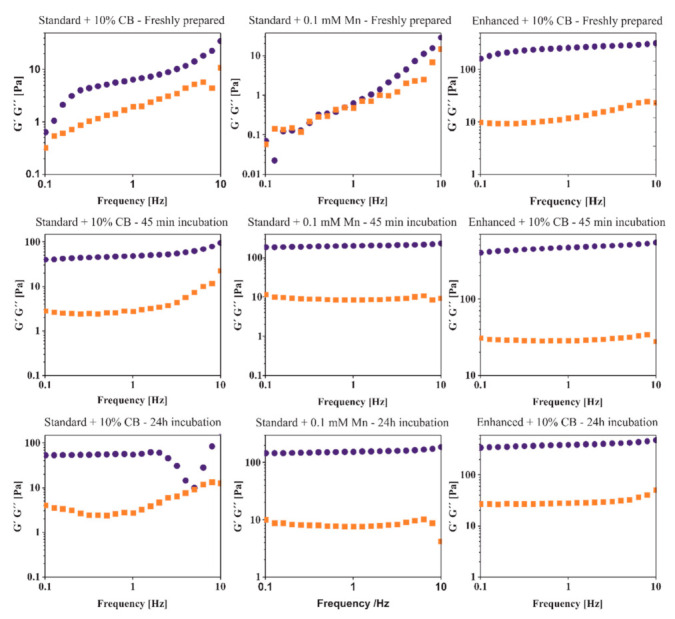
Rheological analysis of the different alginate hydrogels, with/without manganese addition, and different calcium alginate particle concentrations. The analysis was performed immediately after hydrogel formation (upper row), following 45-min incubation with aCSF (middle row) and after 24-h incubation with aCSF (bottom row)—data presented as mean, *n* = 3; orange squares—G” shear modulus (viscous component) (Pa), blue dots—G’ shear modulus (elastic component) (Pa).

The rheological analysis was performed through frequency sweeps (from 0.1 to 10 Hz) of the produced hydrogels to evaluate their mechanical properties. Whether those properties are altered by the presence/absence of compact manganese alginate particles and by the different CaM concentrations (0.5% and 1%) was also assessed. Analyzing ionic cross-linking at short time points was impossible because the cross-linking process already started in the syringe set. Therefore, to assess the effect of CSF on the hydrogels’ mechanical properties, three types of hydrogels were tested in different conditions: freshly prepared; hydrogel incubated with artificial cerebrospinal fluid (aCSF) for 45 min; hydrogel incubated in the presence of aCSF for 12 h. The hydrogels containing the higher concentration of CaM had increased stability and strength, related to a stronger and tighter cross-linked network. However, the addition of compact manganese alginate particles caused a decrease in hydrogel’s stability and strength, displayed in the higher values of elastic component (G’) (Figure 1). In all types of hydrogels, the values of G’ increased considerably, reaching values above 50 Pa in the case of SD + 10% CB and exceeding 100 Pa in two other hydrogel combinations, meaning the stability and strength of all hydrogels increased after 45 min of incubation with aCSF. Furthermore, the breakage/fracture around 5 Hz appearing for SD + 10% CB hydrogel at 24 h indicates that the hydrogel started losing its stability.

The overnight incubation of hydrogels with aCSF slightly increases the stability of hydrogels compared to the freshly prepared hydrogels. Nevertheless, the visible effect was less noticeable than that observed after 45 min of incubation.

### 2.2. Injectability Analysis of LVM

Rheological analysis shows that the hydrogels incubated with aCSF were more stable and stronger. Taking this into account, the injectability analysis was performed for hydrogels, freshly prepared, after 45 min incubation with aCSF (Hamilton syringe) and after 24 h incubation with aCSF (tuberculin syringe). The results of the experiment using the Hamilton syringe showed that the force needed to inject SD + 10% CB hydrogel into aCSF was comparable with the force required to inject water into aCSF. After incubating hydrogel with aCSF for 45 min, a slight increase in force can be observed, according to the rheological analysis. The freshly prepared hydrogels of SD and SD + 10% beads injected with the Hamilton syringe required lower force, reaching values near those of the force needed to inject water. 

On the other hand, a decrease in the force needed to inject freshly prepared SD hydrogel or freshly prepared EN + 10% CB was observed compared to the force required for water injection.

**Figure 2:**
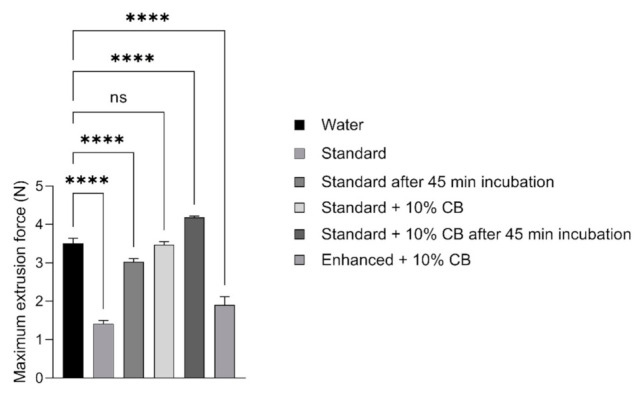
Injectability analysis of alginate-based hydrogels: Extrusion force needed to inject the hydrogels (SD and SD + 10% CB) into the artificial cerebrospinal fluid, using Hamilton syringe with 31G needle. The hydrogels were measured freshly prepared and after 45 min incubation in aCSF. Data presented as mean (±SD), *n* = 2. **** *p* < 0.0001, ns—not significant, compared to water.

After 45 min of hydrogel incubation with aCSF, the force required to inject SD hydrogel increased slightly. Still, it was lower than the force needed to inject water, which is incongruous (Figure 2). In the case of EN + 10% CB hydrogel, its loading into the Hamilton syringe after 45 min incubation was not possible.

**Figure 3:**
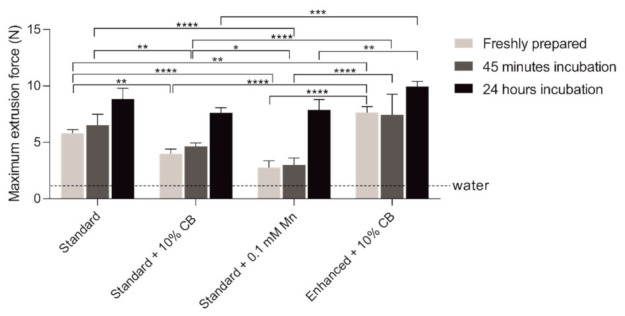
Injectability analysis of alginate-based hydrogels: Extrusion force needed to inject the different hydrogels’ formulations into aCSF, using tuberculin (1 mL) syringe with 27G needle. The hydrogels were measured freshly prepared and after 45 min incubation with aCSF. Data presented as mean (±SD), *n* = 3. * *p* < 0.05; ** *p* < 0.01; *** *p* < 0.001; **** *p* < 0.0001 compared to water.

Due to several obstacles found during the analysis of hydrogels using a small diameter Hamilton syringe, we decided to perform additional analysis with a tuberculin syringe and 27G needle. The difficulties were related to the time of hydrogel incubation with aCSF. As mentioned, using a Hamilton syringe and 31G needle, we were not able to analyze hydrogels that were incubated for 24 h with aCSF due to the inability to load the syringe after such a long time of incubation. Moreover, the aperture used for measuring the force needed for hydrogel was not adjusted to the small diameter syringes/needles, and thus the results were not coherent. Therefore, to compare the difference in injectability between diverse materials, we used a 1 mL tuberculin syringe with a 27G needle (Figure 3). The analysis revealed that all hydrogels had a viscous component that grants them an injection force higher than water. Therefore, the 24-h incubation of hydrogel with aCSF required more force during hydrogel infusion. Similarly, more force had to be used to inject the hydrogel incubated with aCSF for 45-min, but to a lesser extent than in the case of 24-h incubation. A higher injection force was observed in the 1% CaM hydrogel injection among the different hydrogels tested.

On the other hand, the minor injection force was required to inject the standard + 0.1 mM Mn combination. As a result, the increase of hydrogel consistency after 24 h incubation with aCSF was the highest of all tested hydrogels (Figure 1). Most importantly, the primary statistical differences were observed between freshly prepared hydrogels, but these differences decreased after 24-h incubation, related to an increase in hydrogel’s stability and strength.

### 2.3. Permeability Analysis of LVM

**Figure 4:**
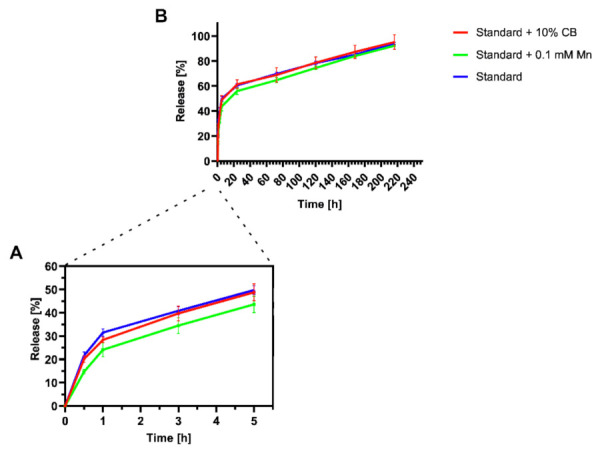
Permeability analysis of alginate hydrogels: (**A**) The release profile of dextran 70 kDa conjugated with FITC from SD hydrogel with compact manganese alginate particles/Mn^2+^/without manganese addition during the first 5 h; and (**B**) The release profile of dextran 70 kDa conjugated with FITC released from SD, SD + 10% CB or SD + 0.1 mM Mn^2+^ hydrogels for the following days. Data presented as mean (±SD), *n* = 3.

To assess the ability of hydrogels to release molecules, permeability analysis was performed for 70-kDa dextran-fluorescein (FITC). As depicted in Figure 4, a similar release profile was observed for hydrogels containing and not containing manganese. It also showed that the different forms of manganese (Mn ions or Mn compact particles) present in hydrogels had no significant impact on their ability to release the dextran particles. In our studies, around 50% of the release was completed within 5 h (Figure 4A). In the following days, continuous release of dextran was observed, gradually reaching almost 100% after 220 h (Figure 4B), showing the potential for sustained and progressive distribution of particles outside of hydrogel structure and proving its permeability for substances with a molecular weight up to 70 kDa.

### 2.4. Analysis of Morphology and Chemical Composition of LVM

**Figure 5:**
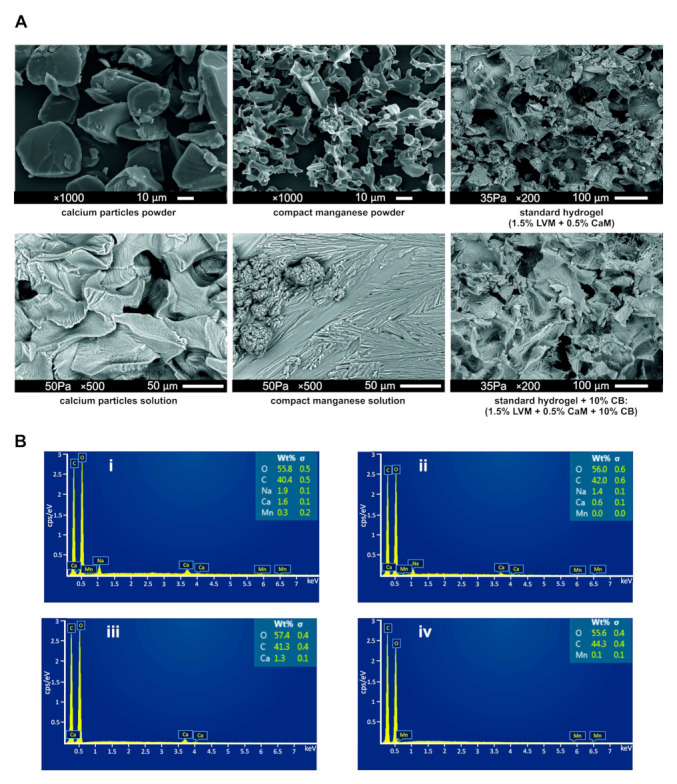
Characteristics of alginate hydrogels: (**A**) SEM analysis of selected hydrogel’s compounds and hydrogel combinations; and (**B**) EDS analysis presenting a chemical composition of (**i**) SD + 10% CB hydrogel; (**ii**) SD hydrogel; (**iii**) calcium alginate powder; (**iv**) compact manganese alginate particles powder.

Morphological structures and chemical composition of different hydrogel combinations (SD/SD + 10% CB) and their components were assessed using scanning electron microscopy (SEM) and Energy Dispersive X-Ray Spectroscopy (EDS). The addition of compact manganese alginate particles slightly changed the architecture of the hydrogels (e.g., pores seem larger), although this is only qualitative analysis (Figure 5A). This trend continued with the remaining tests. In particular, from the rheological studies, the hydrogels were less stable when compact manganese alginate particles were added. Calcium content in both types of hydrogels was low, namely 1.6% for SD + 10% CB and 0.6% for SD (Figure 5(Bi,Bii)). It was enough for hydrogel gelation despite low calcium content and did not cause trouble during the Hamilton syringe loading. It was also noticeable that in SD + 10% compact manganese alginate particles hydrogel, manganese content was low (0.3%). However, it was sufficient to give a signal visible in MRI (Figure 5(Biii,Biv)).

### 2.5. In Vitro Studies

#### 2.5.1. The Assessment of Compact Manganese Particle Impact on Human Adipose-Derived Stem Cells (hADSCs) Viability and Function

**Figure 6:**
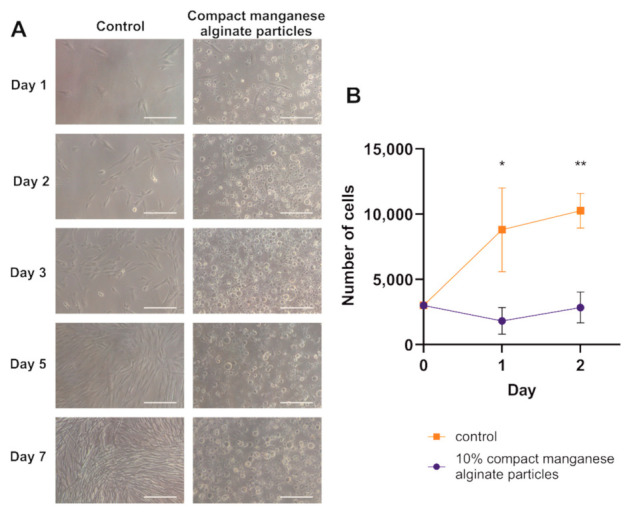
The impact of compact manganese alginate particles on hADSCs cultured in vitro. (**A**) Micrographs of hADSCs cultured with and without the addition of compact manganese alginate particles after 7 days of culturing. Pictures were taken under transmitted light, 100 × magnifications, scale bar—100 µm. (**B**) Cell proliferation assay for hADSCs cultured with and without the addition of compact manganese alginate particles. Absorbance measurement was performed for two consecutive days. Data presented as mean (±SD), *n* = 3. * *p* < 0.05, ** *p* < 0.01 (*t*-student test).

To analyze the impact of compact manganese alginate particles on hADSCs, a seven-day observation of hADSC cultured in a medium containing 10% compact manganese alginate particles was performed. On the 2nd day in vitro (DIV), the apoptotic phenotype was noticed among the hADSC population. No viable cells could be seen in the following days (3–7 DIV), while the control hADCS culture reached 90% confluence on day 7 (Figure 6A). The effect of compact manganese alginate particles on hADSCs proliferation was quantified using a colorimetric assay. Figure 6B shows that the number of hADSCs cultured with compact manganese alginate particles did not change for two consecutive days after cell seeding. At the same time, the proliferation of control hADSCs was noticed. This result suggests that compact manganese alginate particles released from hydrogels may negatively impact stem cells. Unfortunately, this assay was not performed for longer since hADSCs died, so no signal was visible.

#### 2.5.2. Cell Viability Assessment

**Figure 7:**
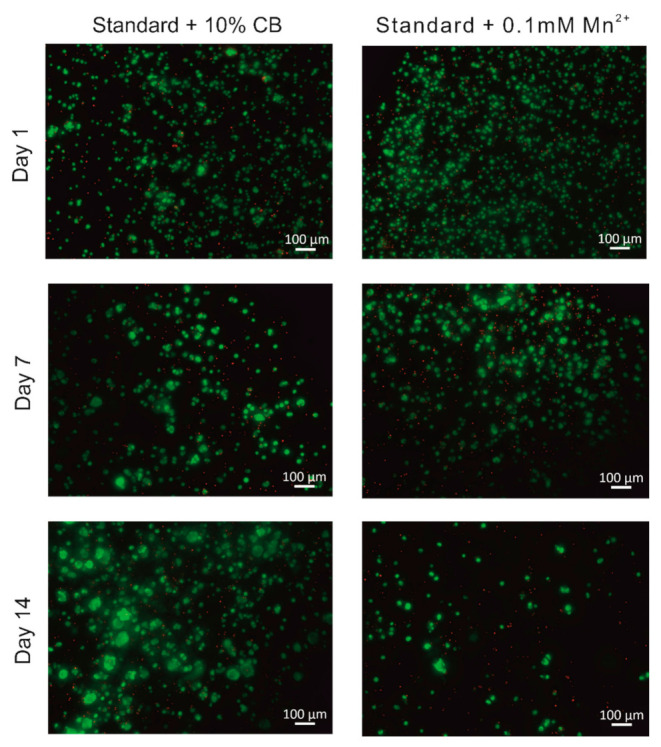
hADSCs encapsulated in alginate hydrogels with/without the addition of manganese. Fluorescent microscopy images of hydrogels extruded from 31G needle were obtained after live/dead staining at 1, 7, and 14 days of culture.

The viability of stem cells in the hydrogel milieu was assessed using hADSCs. The cells were encapsulated in a given hydrogel combination and extruded into aCSF using a 31G needle coupled with a Hamilton syringe. Then aCSF was replaced by culture medium, and hydrogels were incubated for 1, 7, or 14 days. At this particular time point, the viability of hADSCs was determined using fluorescent-based staining (Figure 7). On day 1 a high survival rate was observed, regardless of the manganese presence in ion or compact particles form. The vast majority of cells showed green fluorescence, and very few cells indicated red fluorescence in culture. On day 7, a similar tendency was noticed; again, mainly green fluorescence was detected among cultured hADSCs, and cells showing red fluorescence were a minority.

Interestingly, the aggregation of viable cells was observed in clusters of cells expressing green fluorescence in all types of hydrogels, irrespective of manganese content. Two weeks following hADSC encapsulation, the lowest number of viable cells was seen in a hydrogel containing manganese in ion form. Interestingly at the same time, the number of dead cells in the population seemed to be constant. The tendency to form cell aggregates continued, suggesting that cells were metabolically active, the most prominent in alginate hydrogels supplemented with compact manganese particles. More hADSCs showing green fluorescence were also observed in hydrogels supplemented with CBs.

#### 2.5.3. Magnetic Resonance Imaging of LVM Hydrogel Phantoms

**Figure 8:**
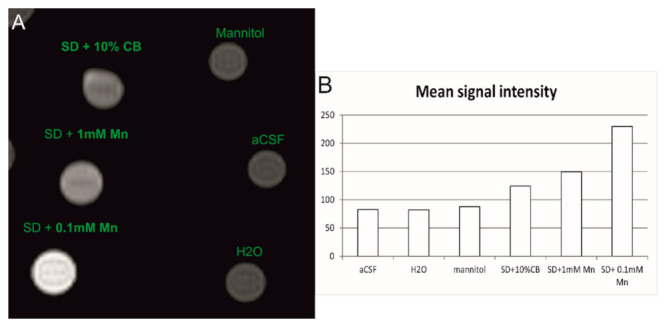
T1-weighted MR images of hydrogel phantoms containing 1 mM and 0.1 mM Mn^2+^ and compact manganese alginate particles (**A**). Mean MR signal intensity measured with the same region of interest (ROI) size (**B**). Mannitol, aCSF and H_2_O was used as a control signal during T1-weighted MR imaging.

Hydrogels labeled with manganese ions (Figure 8) showed a hyperintense signal on T1 MRI. Two manganese ion concentrations were compared and 0.1 mM Mn^2+^ presented with much higher hyper-intensity than 1 mM Mn^2+^, control probes (aCSF, mannitol, or H_2_O), or manganese within compact beads. Hydrogels with 10% compact manganese alginate particles also showed a higher MRI signal than control phantoms. Nevertheless, the signal is less intense than the signal evoked by hydrogels containing ionic Mn^2+^.

#### 2.5.4. MR Imaging of Intrathecally Transplanted LVM Hydrogels

**Figure 9:**
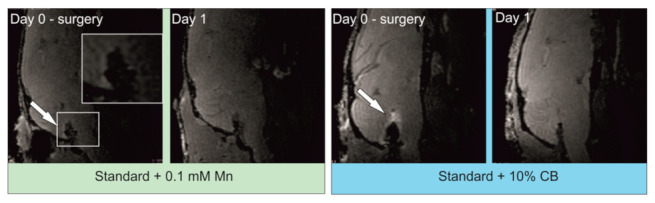
T1-weighted MRI of alginate hydrogels supplemented with 0.1 mM Mn^2+^ or compact manganese alginate particles directly and 24 h after intrathecal transplantation. Arrow indicates a hyperintense signal that corresponds to hydrogel localization. A cropped image of the selected region of interest presents the magnification of the intrathecal area with a hyperintense signal. No signal can be observed after 24 h.

Visualization of alginate hydrogels after their transplantation was confirmed by T1-weighted MR images (Figure 9). Hydrogels with the addition of either 0.1 mM Mn^2+^ or compact manganese particles were injected intrathecally, and T1-weighted MRI scans were performed directly after the surgical procedure and 24 h after the transplantation. Images obtained directly after the hydrogel injection showed the opposite tendency compared to hydrogel phantoms. Higher signal intensity was observed for hydrogels containing compact manganese particles, while hydrogels with ion manganese addition showed significantly lower signal intensity. The MR images obtained 24 h following surgery failed to detect any T1 Mn signal, which was true for both manganese formulations.

## 3. Discussion

In the present work, we developed alginate-based hydrogels that might be applicable in cell transplantation, particularly in stem cell therapy of neurodegenerative diseases such as ALS, where intrathecal delivery is considered. First, we established the appropriate hydrogel composition in terms of its physical properties. Rheological analysis of LVM suggests that the stability of hydrogels increases with an increase in the amount of added calcium ions. Moreover, it seems that the best way to achieve appropriate hydrogel stability, without the danger of a syringe clog, is to pre-incubate the hydrogel in aCSF. Ions present in aCSF may affect the gelation process of hydrogels by contributing to the cross-linking process, increasing their stability. It seems that calcium ions as a cross-linker greatly influence hydrogel stability and permeability, but they also can positively affect the viability of the cells [24,25]. We and others have shown that the mechanical parameters of hydrogel largely depend on the number of bivalent ions and the time of incubation [26]. However, it must be stressed that too many calcium ions may negatively impact the viability of encapsulated cells [27]. Thus, one must carefully balance the amount of cross-linker to assure the desired mechanical properties of hydrogel for a particular purpose, for example, transplantation site, while not affecting the viability of cells embedded in the hydrogel. Since we established that pre-incubation with aCSF influences the rigidity of the hydrogel, we wanted to be sure that the injectability of the alginate scaffold is not affected. Fortunately, pre-incubation of hydrogel with aCSF controlled injectability—the force needed to push the hydrogel through the syringe was slightly higher—however, pre-incubation of alginate scaffold with aCSF did not cause a needle clog. The increase of calcium concentration to 1% disabled hydrogel injection; therefore, we used 0.5% calcium concentration in our subsequent experiments.

Several therapeutic advantages of stem cell treatment are related to their paracrine abilities. Lately, this type of cell has been of great interest to researchers and clinicians due to the different regenerative factors released by cells transplanted to injured tissues [28]. Hence, hydrogels being used as the carriers of exogenous cells should have good permeability to the molecules. Moreover, hydrogels can be supplemented with proteins, that is, growth factors that might enhance the therapeutic effect of grafted cells. Therefore, it is vital to investigate the permeability of hydrogels and verify how fast the potential proteins can be released from them. A recent study from the Mazzon group has shown that human adipose-derived MSCs transplanted into a mouse model of ALS released proteins, that is, BDNF, IGF, NGF, or VEGF, and positively influenced the course of disease [29]. The molecular weight of the most critical growth factors is below 70 kDa. Thus, we used dextran 70 kDa to evaluate the permeability of hydrogels analyzed in our studies. The analysis has shown the satisfactory ability of alginate hydrogels to release proteins up to 70 kDa. Furthermore, the whole process was relatively quick at the beginning—within the first five hours, 50% of dextran had already diffused, and it took around 9 days to unfetter the protein completely. We, therefore, assume that the growth factors released by cells encapsulated in hydrogel could easily penetrate the scaffold and reach the endogenous target after transplantation.

The structure of alginate hydrogels studied by our group does not aberrate from other alginate-based hydrogels [30,31]. Namely, a porous hydrogel structure enables cell settlement and allows the transport of nutrients and the export of growth factors and cytokines released by cells embedded within the scaffold. Calcium alginate beads have influenced pore size; however, we are convinced that it would not negatively affect the entire hydrogel structure. The EDS analysis confirmed the presence of a small amount of calcium and manganese ions. Even a small volume of calcium ions can initiate the gelation process. Our previous work established the amount of manganese needed for MRI visualization [13]. Although manganese is presently used as a contrast agent during manganese-enhanced MR imaging, it is known that excessive accumulation might be harmful to the central nervous system and cause adverse neurological effects [32].

Nevertheless, the toxic effect of manganese accumulation might be diminished by using fractionated dosages and/or nanoparticles with slow Mn^2+^ release [33]. Thus, amounts of manganese ions must be carefully balanced as too high a dose of manganese may not only excessively reduce the relaxation time, preventing the visible signal, but may also be toxic for encapsulated cells [13,34,35]. We have tested the cytotoxicity of manganese ions added directly to the cell medium (data not shown). The addition of 1 mM manganese resulted in a dramatic decrease in hADSC viability, as shown in the MTS test. Interestingly, 0.1 mM manganese present in the culture medium decreased the viability of cells at the beginning of the observation. However, within 7 days of culture, the cells remained constant. The first part of our work concentrated on the optimal hydrogel composition. In the next step, we established the potential impact of alginate hydrogel on the encapsulated cells and their viability in vitro. We encapsulated hADSCs in alginate scaffolds and cultured them in hydrogel for up to 14 days. As the hydrogel structures are very delicate and the medium change was sometimes difficult due to the small scaffolds (10 uL) and their transparency, we cultured hADSCs on the membranes. The cells were viable throughout the entire observation time. However, we observed an increase in the number of dead cells as the culture extended. We speculate that this process is quite natural because even though the cells are encapsulated as a mixture, they are separated by the hydrogel’s structure. In standard culture conditions, MSCs need the optimal density and cell contacts; thus, the separation by the hydrogel is not their natural state that would increase the proliferation [36]. Three dimensional (3D) cell culture can influence MSC proliferation; however, the positive or negative effect on cell division largely depends on applied cell culture protocols [37,38].

The possibility of using the proposed MRI technique to visualize the localization of transplanted cell-laden hydrogels is of great importance for understanding and boosting the translation of stem cell therapeutic applications into the clinical setting. We have previously reported using manganese as a contrast agent for hydrogel visualization [13]. Here, we have tested and compared manganese in ions and compact manganese alginate particles. The application of CaM was prompted by the idea of possible MR signal intensification or prolongation of the possible visualization time. Phantom MR imaging allowed us to determine the proper manganese concentration both of ions and compact beads. Phantom imaging revealed a higher signal intensity related to manganese ions than compact manganese beads. However, at lower levels of the probe, CBs were more apparent.

We think that the dispersion of ions within the hydrogels might have been slightly better, causing prominent visibility. Compact beads might have settled with gravity during the scanning procedure. On the contrary, after transplantation of the hydrogels into the intrathecal space, those containing CBs were more clearly visible. The diffusivity of manganese was previously described by Liu and co-workers in general [39]. The manganese dispersed through the ventricular system and diffused into the brain parenchyma. Depending on manganese concentration, the intense signal was visible as far as in the olfactory bulb up to the brain stem. In comparison with our results, the diffusion observed by Liu and co-workers was much higher and more intense [39]. However, the manganese concentration used by the authors was much higher than that used in our studies. Moreover, in our experiments, the signal dispersion was visible only around the brain stem and medulla. This might be related to the low manganese concentration and the fact that ions and manganese CB were dissolved in a hydrogel, which probably restricted diffusion. Recently, Cha and others presented the results of MRI analysis after intrathecal manganese injection. The authors observed signal intensification within the spinal cord of experimental rats [40]. It is also reasonable to consider that some manganese/CB also might have dispersed toward the caudal direction. However, we did not perform an MRI of the spinal cord, and future studies in this direction will be carried out. Both manganese ions and CBs were not visible 24 h after transplantation. We hoped that manganese compact bead application would increase the imaging time. However, it seems that the diffusion of manganese in both cases is relatively fast, disabling its visualization over a more extended period. Nevertheless, the results of our studies confirmed that the location assessment of hydrogels in the host tissue after their transplantation is possible via MR imaging.

## 4. Materials and Methods

### 4.1. Preparation of Mannitol and LVM Solutions, CaM Solution with Compact Manganese Alginate Particles or MnCl_2_, and aCSF Solution

Mannitol powder (Sigma-Aldrich, St. Louis, MO, USA) was dissolved in Milli-Q water to obtain a 4.6% mannitol solution. Low viscosity sodium alginate LVM and calcium alginate (CaM) solutions were prepared by dissolving LVM/CaM powder (Novamatrix, Sandvika, Norway) in a 4.6% mannitol solution with a magnetic field stirrer used for agitation. Manganese (II) chloride powder (Sigma-Aldrich, St. Louis, MO, USA) was dissolved in Milli-Q water to acquire MnCl_2_ aqueous solution. Calcium alginate solution was supplemented with either MnCl_2_ or compact manganese alginate particles (Novamatrix, Sandvika, Norway). aCSF was prepared as in [13]. All solutions were sterilized before in vitro experiments using 0.2 μm filters, and calcium alginate as CaM powder was sterilized by UV light before solution preparation.

### 4.2. Preparation of Alginate Hydrogels

Alginate hydrogels formulations were obtained in a syringe set using a 1.5% LVM solution with a 0.5% or 1% CaM solution. This solution was supplemented with 0.1 mM MnCl_2_ or compact manganese alginate particles (CB) powder to acquire a 10% concentration. To simulate in vivo conditions as well as to intensify the cross-linking process, hydrogels were prepared in molds, and aCSF drops were added. 

### 4.3. Rheological Analysis

The rheological analysis was performed using a Kinexus Pro+ Rheometer (Malvern Panalytical, Malvern, UK) with acquisition software rSpace (NETZSCH-Gerätebau, Selb, Germany). A parallel plate system with an upper plate diameter 8 mm and a lower plate diameter 20 mm; the 1-mm gap between plates was used for oscillation experiments. Frequency sweep measurements were performed for 30 min with a start frequency of 0.1 Hz, an end frequency of 10 Hz, and a shear strain of 0.5% at 37 °C. Different types of hydrogels, with or without compact manganese alginate particles and 1% or 0.5% CaM concentration, were tested in three conditions: freshly prepared, after 45 min, or overnight incubation with aCSF (as intrathecal delivery of hydrogels into the CSF will be performed). The hydrogel solution was placed at a lower plate, and measurement was conducted with three repetitions.

### 4.4. Injectability Analysis

#### 4.4.1. Hamilton Syringe (31G)

The injectability analysis was performed using a specific set of equipment (Paralab, Valbom, Portugal; KD Scientific, Holliston, MA, USA) and Hamilton syringe 1700, 10 µL volumes with a 31G needle (inner diameter—0.133 mm). The Hamilton syringe was loaded with freshly prepared hydrogel or hydrogel incubated for 45 min with aCSF, and injection was made into aCSF with a constant speed of 10 µL/minute. The force required for the injection procedure was captured and displayed by the software provided by the manufacturer. Injection of water into aCSF was used as a control. At least three repetitions were performed for each condition and control.

#### 4.4.2. Tuberculin Syringe (27G)

The injectability analysis was performed as described above; however, using a 27G needle (inner diameter—0.21 mm) with a 1 mL tuberculin syringe [*Henke*-Sass Wolf (*HSW*), Tuttlingen, Germany]. The analysis was made with freshly prepared hydrogel, after 45 min incubation with aCSF and after the 24-h incubation with aCSF.

### 4.5. Permeability Analysis

Hydrogels’ permeability was analyzed using dextran with molecular weight 70 kDa conjugated with FITC (Thermo Fisher Scientific, Waltham, MA, USA). Dextran-FITC solution was prepared and combined with a 1.5% LVM solution to reach a final Dextran-FITC concentration of 125 µg/mL. Subsequently, hydrogel formulations were obtained as described above and placed in aCSF solution with shaking at 37 °C. At given time points, 350 µL of aCSF solution was taken and replaced with fresh aCSF solution, at the last time point hydrogels were mechanically destroyed, and 350 µL of aCSF solution was taken. Fluorescence was measured using the FLUOstar Omega microplate reader (BMG LABTECH, Ortenberg, Germany) (excitation wavelength 485 nm, emission wavelength 528 nm) to calculate the amount of released Dextran-FITC into aCSF. The standard curve was prepared using solutions with a known Dextran-FITC concentration for calculations.

### 4.6. Analysis of LVM Morphology and Chemical Composition

LVM morphology and chemical composition were analyzed for hydrogel formulations (1.5% LVM, 0.5% CaM with or without compact manganese alginate particles), CaM solution and powder, compact manganese alginate particles solution, and powder. Hydrogel formulations were placed at −80 °C and, after 48 h, lyophilized (LyoAlfa 10/15, Telstar, Terrassa, Spain). Dried hydrogel formulations, CaM, and compact manganese alginate particles solutions and powders were glued to the mounting using carbon tape. The analysis of morphology and EDS for chemical composition analysis was performed using SEM (JEOL—JSM-6010 LV, Jeol, Tokyo, Japan).

### 4.7. In Vitro Studies

#### 4.7.1. Cell Culture

hADSCs were isolated from adipose tissue obtained during liposuction from the abdominal region after informed consent from the patient (Medical University of Warsaw Bioethical Committee permission). The obtained cells were cultured as monolayers in commercially available medium RoosterBasalTM-MSC (RoosterBio, Frederick, MD, USA) and passaged after reaching 80–90% confluence. Then the cells were detached using TrypLE^TM^ Express (Thermo Fisher Scientific, Waltham, MA, USA), centrifuged at 1000 rpm for 5 min, counted, and seeded on the culture dishes at the density of 2000–3000/cm^2^. For in vitro studies, hADSCs at passages 4–6 were used.

#### 4.7.2. Compact Manganese Alginate Particles Toxicity Assessment

Standard curve preparation: hADSCs at passages 4–6 were seeded in 100 µL of culture medium to 96-well plate in following densities: 250, 500, 750, 1000, 2000, 5000, 10,000, 15,000 or 25,000/cm^2^. After 24 h, 10 µL of WST-8 solution (The Cell Counting Kit 8; Dojindo, Kumamoto, Japan) was added to each well and incubated for 2 h at 37 °C. The absorbance was measured at optical density (OD) 450 nm using the FLUOstar Omega microplate reader (BMG LABTECH, Ortenberg, Germany).

hADSCs at passages 4–6 were seeded in 100 µL of culture medium with compact manganese alginate particles at 10% concentration or without (control) 96-well plate in density 3000 cells/cm^2^. After 24 or 48 h, 10 µL of the WST-8 solution was added to each well and incubated for 2 h at 37 °C. The absorbance was measured at OD 450 nm using the FLUOstar Omega microplate reader (BMG LABTECH, Ortenberg, Germany).

#### 4.7.3. Encapsulation of hADSC Cells

hADSCs at passages 4–6 were detached from cell culture flask using TrypLE™ Express (Thermo Fisher Scientific, Waltham, MA, USA) and centrifuged at 1000 rpm for 5 min. First, the cellular pellet was suspended in a hydrogel formulation. Three hydrogel formulations were used for cell encapsulation: SD + 10% CB or SD + 0.1 mM MnCl_2_ and SD without manganese addition. The final cell density in hydrogel formulation was 2 × 10^6^/mL. Then, 10 µL of hydrogel with encapsulated cells was introduced into a Hamilton syringe 1700. The mixture injection was made into aCSF with a 10 µL/minute rate using a Quintessential Stereotaxic Injector pump (QSITM, Stoelting Co. Ltd., Wood Dale, IL, USA). After 45 min, aCSF was replaced by a culture medium. Next, hydrogels (volume = 10 µL) were cultured for 1, 7, or 14 days in organotypic cell culture inserts (Millicell^®^, Merck Millipore, Burlington, MA, USA) in 6-well plates with 900 µL of culture medium. For each time point, a separate plate was prepared.

#### 4.7.4. Cell Viability Assessment

At given time points (1, 7, or 14 days), cell viability was assessed using fluorescence-based LIVE/DEAD^TM^ Viability/Cytotoxicity Kit for mammalian cells (Thermo Fisher Scientific, Waltham, MA, USA); Calcein dye is maintained in living cells giving green fluorescence. In contrast, ethidium homodimer-1 in cells with damaged membranes shows red fluorescence upon nucleic acid binding. Calcein AM and Ethidium homodimer-1 were suspended in sterile PBS (2 µL of Ethidium homodimer-1, 0.5 µL of Calcein-AM in 1 mL of PBS), and 200 µL of the solution was added per well, followed by 20 min of incubation at room temperature (RT). The images were collected using a Cell Observer SD microscope (Axio Observer Z.1, Carl Zeiss, Jena, Germany) in Z-stack mode with an interval of 2.13 µm. Filter excitation/emission wavelength 488/509 (green channel) and 545/572 (red channel) were used. Ranges of emission: 450–490, 500–550, 538–562, 570–640. Image size in pixels: 1388 × 1040 with the scaling (per pixel) 1024 × 1024. Obtained images were analyzed using Zeiss ZEN (Carl Zeiss, Jena, Germany) and ImageJ software (v.1.53o).

#### 4.7.5. Magnetic Resonance Imaging of LVM Hydrogel Phantoms

Hydrogels were prepared as described above. Eppendorf tubes (1.5 mL volume) containing different solutions of LVM hydrogels, water, or aCSF as control were placed in an MRI scanner (BioSpec 70/30 USR, Bruker, Ettlingen, Germany). T1-weighted 2D FLASH sequence (repetition time, TR = 180 ms; echo time, TE = 7.2 ms; flip angle, FA = 30°; the number of averages, NA = 1; field of view, FOV = 80 mm × 80 mm; spatial resolution = 312 µm isotropic, slice 2 mm; acquisition time, TA ~45 s) was used. A signal from each phantom was encircled with an identical outline comprising ROI. Evaluation of signal intensity from each ROI was made using ImageJ software (v.1.53o).

### 4.8. In Vivo Studies

#### 4.8.1. Intrathecal Transplantation of LVM Hydrogel

The animal procedures were performed with the approval of the IV Local Committee in Warsaw, 117/2015. Animals were anesthetized with isoflurane and placed in the stereotaxic frame in a Concorde-like position [41]. A small vertical skin incision of approximately 4–5 mm was made directly under the line of the skull end. Soft tissues were delicately moved aside, exposing the atlanto-occipital membrane. LVM hydrogels for transplantation were prepared as described above. Two hydrogel components were mixed directly before the injection and loaded in a Hamilton syringe 1700. The syringe was placed in a stereotaxic injector so that the needle would intersect the atlanto-occipital membrane under the right angle to its surface. Hydrogel infusion (10 µL) into the intrathecal space was made with a constant speed of 10 µL/min. The needle was slowly withdrawn to avoid the leakage of aCSF and hydrogel approximately two minutes after injection was finished. Lastly, the skin was sutured, and the animals were subjected to MR imaging (see below).

#### 4.8.2. In Vivo MR Imaging

Directly after the surgery, the animals were transferred to an MRI-compatible water-heated bed. Body temperature and respiration rate were measured throughout the imaging procedure. T1-weighted 3D FLASH sequence (TR = 12 ms, TE = 4 ms; FA = 18, NA= 10, FOV = 15 mm × 15 mm, spatial resolution = 117 µm isotropic, slice 1 mm, TA ~ 25 min) was used. The animals also were subjected to MR scanning 24 h after the first scan. Mice were anesthetized with isoflurane, and the scanning procedure was repeated exactly in the same conditions. All the scanning procedures were performed using a 7T MR scanner (BioSpec 70/30 USR, Bruker, Ettlingen, Germany) equipped with a transmit cylindrical radiofrequency coil (8.6 cm inner diameter, Bruker, Ettlingen, Germany) and a mouse brain dedicated receive-only array surface coil (2 × 2 elements, Bruker, Ettlingen, Germany).

### 4.9. Statistical Analysis

Results are presented as mean ± SD. Statistical analysis was conducted using GraphPad Prism 9 software (San Diego, CA, USA). Kruskal–Wallis and t-student tests were performed when applicable. *p*-values less than 0.05 were considered statistically significant.

## 5. Conclusions

Our studies analyzed and optimized the properties of alginate-based hydrogels that might be suitable as cell carriers used in regeneration therapy. These hydrogels can provide a microenvironment for embedded cells, that is, adequate physical and chemical conditions, creating a unique matrix protecting the encapsulated cells from destructive forces during their injection into the host tissues. Such a scaffold can also insulate the cells from the hostile environment of the recipient directly after their injection. Moreover, the alginate-based hydrogels loaded with manganese ions enable non-invasive MR imaging allowing for hydrogel visualization within the living organism after its implementation. All these features make the alginate-based hydrogels suitable and attractive for parenteral administration as supporting matrices for therapeutic cells.

## Data Availability

The datasets used and/or analyzed during the current study are available from the corresponding author on reasonable request.

## References

[1] Philips T., Mironova Y.A., Jouroukhin Y., Chew J., Vidensky S., Farah M.H., Pletnikov M.V., Bergles D.E., Morrison B.M., Rothstein J.D. (2021). MCT1 Deletion in Oligodendrocyte Lineage Cells Causes Late-Onset Hypomyelination and Axonal Degeneration. Cell Rep..

[2] Golubczyk D., Malysz-Cymborska I., Kalkowski L., Janowski M., Coates J.R., Wojtkiewicz J., Maksymowicz W., Walczak P. (2019). The Role of Glia in Canine Degenerative Myelopathy: Relevance to Human Amyotrophic Lateral Sclerosis. Mol. Neurobiol..

[3] Andrzejewska A., Lukomska B., Janowski M. (2019). Concise Review: Mesenchymal Stem Cells: From Roots to Boost. Stem Cells.

[4] Bonafede R., Mariotti R. (2017). ALS pathogenesis and therapeutic approaches: The role of mesenchymal stem cells and extracellular vesicles. Front. Cell. Neurosci..

[5] Forostyak S., Sykova E. (2017). Neuroprotective potential of cell-based therapies in ALS: From bench to bedside. Front. Neurosci..

[6] Nowak B., Rogujski P., Janowski M., Lukomska B., Andrzejewska A. (2021). Mesenchymal stem cells in glioblastoma therapy and progression: How one cell does it all. Biochim. Biophys. Acta—Rev. Cancer.

[7] Ciervo Y., Ning K., Jun X., Shaw P.J., Mead R.J. (2017). Advances, challenges and future directions for stem cell therapy in amyotrophic lateral sclerosis. Mol. Neurodegener..

[8] Andrzejewska A., Dabrowska S., Nowak B., Walczak P., Lukomska B., Janowski M. (2020). Mesenchymal stem cells injected into carotid artery to target focal brain injury home to perivascular space. Theranostics.

[9] Guzman R., Janowski M., Walczak P. (2018). Intra-Arterial Delivery of Cell Therapies for Stroke. Stroke.

[10] Malysz-Cymborska I., Golubczyk D., Kalkowski L., Kwiatkowska J., Zawadzki M., Głodek J., Holak P., Sanford J., Milewska K., Adamiak Z. (2021). Intra-arterial transplantation of stem cells in large animals as a minimally-invasive strategy for the treatment of disseminated neurodegeneration. Sci. Rep..

[11] Hached F., Vinatier C., Le Visage C., Gondé H., Guicheux J., Grimandi G., Billon-Chabaud A. (2017). Biomaterial-assisted cell therapy in osteoarthritis: From mesenchymal stem cells to cell encapsulation. Best Pract. Res. Clin. Rheumatol..

[12] Oliveira J.M., Carvalho L., Silva-Correia J., Vieira S., Majchrzak M., Lukomska B., Stanaszek L., Strymecka P., Malysz-Cymborska I., Golubczyk D. (2018). Hydrogel-based scaffolds to support intrathecal stem cell transplantation as a gateway to the spinal cord: Clinical needs, biomaterials, and imaging technologies. NPJ Regen. Med..

[13] Vieira S., Strymecka P., Stanaszek L., Silva-Correia J., Drela K., Fiedorowicz M., Malysz-Cymborska I., Rogujski P., Janowski M., Reis R.L. (2020). Methacrylated gellan gum and hyaluronic acid hydrogel blends for image-guided neurointerventions. J. Mater. Chem. B.

[14] Wang J., Wang H., Ramsay I.A., Erstad D.J., Fuchs B.C., Tanabe K.K., Caravan P., Gale E.M. (2018). Manganese-Based Contrast Agents for Magnetic Resonance Imaging of Liver Tumors: Structure-Activity Relationships and Lead Candidate Evaluation. J. Med. Chem..

[15] Ciofani G., Raffa V., Pizzorusso T., Menciassi A., Dario P. (2008). Characterization of an alginate-based drug delivery system for neurological applications. Med. Eng. Phys..

[16] Rahmati M., Ehterami A., Saberani R., Abbaszadeh-Goudarzi G., Rezaei Kolarijani N., Khastar H., Garmabi B., Salehi M. (2020). Improving sciatic nerve regeneration by using alginate/chitosan hydrogel containing berberine. Drug Deliv. Transl. Res..

[17] Manzari-Tavakoli A., Tarasi R., Sedghi R., Moghimi A., Niknejad H. (2020). Fabrication of nanochitosan incorporated polypyrrole/alginate conducting scaffold for neural tissue engineering. Sci. Rep..

[18] Totten J.D., Alhadrami H.A., Jiffri E.H., McMullen C.J., Seib F.P., Carswell H.V.O. (2021). Towards clinical translation of ‘second-generation’ regenerative stroke therapies: Hydrogels as game changers?. Trends Biotechnol..

[19] Levit R.D., Landázuri N., Phelps E.A., Brown M.E., García A.J., Davis M.E., Joseph G., Long R., Safley S.A., Suever J.D. (2013). Cellular encapsulation enhances cardiac repair. J. Am. Heart Assoc..

[20] Allen A.B., Gazit Z., Su S., Stevens H.Y., Guldberg R.E. (2014). In vivo bioluminescent tracking of mesenchymal stem cells within large hydrogel constructs. Tissue Eng.—Part C Methods.

[21] Hosseini S.M., Sharafkhah A., Koohi-Hosseinabadi O., Semsar-Kazerooni M. (2016). Transplantation of neural stem cells cultured in alginate scaffold for spinal cord injury in rats. Asian Spine J..

[22] Abasalizadeh F., Moghaddam S.V., Alizadeh E., Akbari E., Kashani E., Fazljou S.M.B., Torbati M., Akbarzadeh A. (2020). Alginate-based hydrogels as drug delivery vehicles in cancer treatment and their applications in wound dressing and 3D bioprinting. J. Biol. Eng..

[23] Kalkowski L., Golubczyk D., Kwiatkowska J., Holak P., Milewska K., Janowski M., Oliveira J.M., Walczak P., Malysz-Cymborska I. (2021). Two in One: Use of Divalent Manganese Ions as Both Cross-Linking and MRI Contrast Agent for Intrathecal Injection of Hydrogel-Embedded Stem Cells. Pharmaceutics.

[24] Golmohamadi M., Wilkinson K.J. (2013). Diffusion of ions in a calcium alginate hydrogel-structure is the primary factor controlling diffusion. Carbohydr. Polym..

[25] Sarker M., Izadifar M., Schreyer D., Chen X. (2018). Influence of ionic crosslinkers (Ca^2+^/Ba^2+^/Zn^2+^) on the mechanical and biological properties of 3D Bioplotted Hydrogel Scaffolds. J. Biomater. Sci. Polym. Ed..

[26] Naghieh S., Karamooz-Ravari M.R., Sarker M.D., Karki E., Chen X. (2018). Influence of crosslinking on the mechanical behavior of 3D printed alginate scaffolds: Experimental and numerical approaches. J. Mech. Behav. Biomed. Mater..

[27] Cao N., Chen X.B., Schreyer D.J. (2012). Influence of Calcium Ions on Cell Survival and Proliferation in the Context of an Alginate Hydrogel. ISRN Chem. Eng..

[28] Lukomska B., Stanaszek L., Zuba-Surma E., Legosz P., Sarzynska S., Drela K. (2019). Challenges and Controversies in Human Mesenchymal Stem Cell Therapy. Stem Cells Int..

[29] Gugliandolo A., Bramanti P., Mazzon E. (2019). Mesenchymal stem cells: A potential therapeutic approach for amyotrophic lateral sclerosis?. Stem Cells Int..

[30] Nita L.E., Chiriac A.P., Ghilan A., Rusu A.G., Tudorachi N., Timpu D. (2021). Alginate enriched with phytic acid for hydrogels preparation. Int. J. Biol. Macromol..

[31] Distler T., Lauria I., Detsch R., Sauter C.M., Bendt F., Kapr J., Rütten S., Boccaccini A.R., Fritsche E. (2021). Neuronal Differentiation from Induced Pluripotent Stem Cell-Derived Neurospheres by the Application of Oxidized Alginate-Gelatin-Laminin Hydrogels. Biomedicines.

[32] Silva A.C., Bock N.A. (2007). Manganese-Enhanced MRI: An Exceptional Tool in Translational Neuroimaging. Schizophr. Bull..

[33] Bock N.A., Paiva F.F., Silva A.C. (2008). Fractionated Manganese-Enhanced Magnetic Resonance Imaging. NMR Biomed..

[34] Tambalo S., Daducci A., Fiorini S., Boschi F., Mariani M., Marinone M., Sbarbati A., Marzola P. (2009). Experimental protocol for activation-induced manganese-enhanced MRI (AIM-MRI) based on quantitative determination of Mn content in rat brain by fast T1 mapping. Magn. Reson. Med..

[35] Ding D., Roth J., Salvi R. (2011). Manganese is toxic to spiral ganglion neurons and hair cells in vitro. Neurotoxicology.

[36] Drela K., Stanaszek L., Nowakowski A., Kuczynska Z., Lukomska B. (2019). Experimental strategies of mesenchymal stem cell propagation: Adverse events and potential risk of functional changes. Stem Cells Int..

[37] Bogdanova-Jatniece A., Berzins U., Kozlovska T. (2014). Growth properties and pluripotency marker expression of spontaneously formed thre-dimensional aggregates of human adipose-derived stem cells. Int. J. Stem Cells.

[38] Nikolits I., Nebel S., Egger D., Kreß S., Kasper C. (2021). Towards Physiologic Culture Approaches to Improve Standard Cultivation of Mesenchymal Stem Cells. Cells.

[39] Liu C.H., D’Arceuil H.E., De Crespigny A.J. (2004). Direct CSF Injection of MnCl2 for Dynamic Manganese-Enhanced MRI. Magn. Reson. Med..

[40] Cha M., Lee K., Won J.S., Lee B.H. (2019). Manganese-enhanced magnetic resonance imaging of the spinal cord in rats with formalin-induced pain. Neurosci. Res..

[41] Janowski M., Kuzma-Kozakiewicz M., Binder D., Habisch H.J., Habich A., Lukomska B., Domanska-Janik K., Ludolph A.C., Storch A. (2008). Neurotransplantation in mice: The concorde-like position ensures minimal cell leakage and widespread distribution of cells transplanted into the cisterna magna. Neurosci. Lett..

